# Intravenous Steroids for Refractory Chemotherapy-Related Diarrhea: A Case Report

**DOI:** 10.7759/cureus.34634

**Published:** 2023-02-04

**Authors:** Kishan Patel, Adrian Dang, Mai M Makhlouf, Alex G Raman, Nihal E Abdulla

**Affiliations:** 1 College of Osteopathic Medicine of the Pacific, Western University of Health Sciences, Pomona, USA; 2 Department of Oncology, Los Alamitos Medical Center, Los Alamitos, USA

**Keywords:** case report, internal medicine, oncology, chemotherapy-related diarrhea, intravenous steroids

## Abstract

The clinical course of a patient with chemotherapy-related diarrhea (CRD) refractory to standard therapy was monitored over the course of 21 days. The patient was minimally responsive to traditional treatment options, including bismuth subsalicylate, diphenoxylate-atropine, loperamide, octreotide, and oral (PO) steroids, and exhibited reportable improvements with the addition of intravenous (IV) methylprednisolone to other antidiarrheal agents. We present a case of CRD in an 82-year-old female. She was initiated on chemotherapy three weeks prior and has experienced severe diarrhea since her initiation. Despite the use of first-line antidiarrheal therapies, including loperamide, diphenoxylate-atropine, and octreotide, both subcutaneously and via continuous infusion drip, no infectious cause was found. She also received the non-absorbing corticosteroid budesonide, but her diarrhea persisted. After experiencing severe hypotension and hypovolemia secondary to profuse diarrhea, she was placed on IV steroids, which quickly reduced her symptoms. The patient was then transitioned to oral steroids and discharged on a tapering regimen. We recommend using IV steroids to treat CRD if first-line therapies fail. Utilizing IV steroids efficiently and effectively can decrease the symptoms of persistent diarrhea and lead to rapid recovery.

## Introduction

Chemotherapy-related diarrhea (CRD) is a common limiting factor that creates complications in the dosing and administration of chemotherapy [1,2]. In scenarios where other common causes have been ruled out, such as irritable bowel syndrome, malabsorptive syndromes, and infection, chemotherapy can cause diarrhea through secretory, osmotic, or motility-related mechanisms [1]. Chemotherapeutic agents can induce damage to the gastric mucosa's disruptive reabsorptive capacity. This disruption of the mucosa can also alter the expression of digestive enzymes, such as lactase in the brush border, which allow for the accumulation of osmotic agents that can induce diarrhea. Alterations in gastric motility have also been noted, which can cause diarrhea even in the setting of fasting [1].

## Case presentation

Our patient is an 82-year-old female with a past medical history of subcapsular right hepatic hematoma who presented to the emergency department with weakness along with nausea, vomiting, and diarrhea that started after undergoing chemotherapy with trastuzumab deruxtecan three weeks prior. She was previously diagnosed with invasive ductal carcinoma of the left breast, ER/PR+, and low HER2+ disease. Initial physical examination showed an afebrile female with heart rate 106/min, respiratory rate 17/min, and blood pressure 75/35 mmHg. She was in no apparent distress with dry oral mucosa. The chest was clear to auscultation bilaterally, with tachycardia, a soft, nontender abdomen, and no edema. Skin inspection revealed no jaundice. Initial laboratory studies were significant for low serum sodium of 133 mEq/L (reference range: 135 to 145 mEq/L), severely low serum potassium of 2.1 mEq/L (reference range: 3.7 to 5.2 mEq/L), low serum bicarbonate of 20.0 mEq/L (reference range: 23 to 29 mEq/L), elevated serum blood urea nitrogen (BUN) of 58 (reference range: 6 to 20 mg/dL), and elevated serum creatinine of 2.2 (reference range: 0.6 to 1.3 mg/dL). She was managed initially with electrolyte replacement, stool studies, and repeat basic metabolic panel (BMP) studies to treat her diarrhea.

A medical regimen consisting of bismuth subsalicylate around the clock (ATC), diphenoxylate-atropine as needed (PRN), cholestyramine 4 g, PO, three times daily (TID), loperamide 4 mg PO, two caps twice daily (BID), octreotide 150 micrograms (mcg) intravenous (IV) push, every eight hours was initiated. This was then later switched to octreotide infusion and budesonide PO, 9 mg, three caps daily, which was ultimately still insufficient to improve her persistent diarrhea.

Due to our patient’s severe and persistent diarrhea, she experienced severe hypotension and hypovolemia and was upgraded to the intensive care unit (ICU). Management at this juncture was IV norepinephrine at 2 mcg/min, included with albumin human 25% IV piggyback. At this moment, it was decided to initiate this patient on methylprednisolone in an attempt to alleviate the continued diarrhea.

The following day, our patient reported a significant improvement in diarrhea and continued to show improvement over the next few days. Finally, she was placed on oral weaning steroids and discharged.

## Discussion

We describe a case of chemotherapy-related diarrhea (CRD) that persisted despite mainstay treatment therapy. This report details an extensive approach to the management of CRD with the limitation of follow-up and long-term outcomes. Loperamide and diphenoxylate-atropine are widely used for initial therapy for CRD [3,4]. For patients who have diarrhea refractory to loperamide, octreotide is the next-line of treatment [1].

Usage of oral steroids such as budesonide can be considered an effective treatment option in cases of chemotherapy-induced diarrhea that is refractory to first-line measures of diarrhea management [4]. In some cases, as in ours, oral steroids may still not be effective. IV steroids such as methylprednisolone are effective options that can be considered to help reduce diarrhea and minimize the risk of episodic hemodynamic instability. Methylprednisolone, which successfully reduced our patient’s symptoms, works by suppressing the synthesis of cyclooxygenase (COX)-2, which helps to limit the inflammation cascade in damaged tissues due to decreased prostaglandin production [5]. In a separately reported case of mycophenolate-induced colitis, IV methylprednisolone helped to improve symptoms of bloody diarrhea in a patient [6]. In another case, IV methylprednisolone was used to treat diarrhea caused by inflammatory bowel disease [7]. At present, there is little literature on the use of IV steroids for chemotherapy-related diarrhea. However, in our case, IV steroids played a vital role in reducing our patient’s symptoms and hospital stay.

In the medical management of CRD, the consideration of IV steroids should not be postponed. The most efficacious timing of IV steroid administration in the management of CRD is not entirely clear and warrants further clinical investigation. Further research must also be done to minimize the risk of opportunistic infections that can often arise as a result of the immunosuppressive effects of IV steroids. Based on the isolated presentation of our patient's clinical course, it can be implied that the use of IV steroids on the floor could likely decrease the length of stay and improve symptoms more rapidly. There is evidence that IV steroids are beneficial in other cases of persistent diarrhea. In the case of autoimmune enteropathy, IV steroids followed by prednisone 60 mg daily were successfully used to reduce diarrhea, supporting the efficacy of IV steroid usage in the management of persistent diarrhea [8]. Data has also demonstrated that CRD is relatively common in treatments using trastuzumab deruxtecan, with 29% of patients reporting diarrhea [9].

## Conclusions

While IV steroids are not commonly used in the treatment of CRD, clinicians and providers should be aware of their potential benefits in treatment. Based on this case, we recommend the consideration of IV steroids as soon as possible for diarrheal symptoms refractory to first-line therapies and oral steroids.

## References

[1] Krishnamurthi SS, Macaron C (2022). UpToDate. Management of Acute Chemotherapy-Related Diarrhea.

[2] Wadler S, Benson AB 3rd, Engelking C (1998). Recommended guidelines for the treatment of chemotherapy-induced diarrhea. J Clin Oncol.

[3] Lenfers BH, Loeffler TM, Droege CM, Hausamen TU (1999). Substantial activity of budesonide in patients with irinotecan (CPT-11) and 5-fluorouracil induced diarrhea and failure of loperamide treatment. Ann Oncol.

[4] Koselke EA, Kraft S (2013). Chemotherapy-induced diarrhea: options for treatment and prevention. J Oncol Pharm.

[5] Ocejo A, Correa R (2022). Methylprednisolone. StatPearls [Internet].

[6] Farooqi R, Kamal A, Thind K, Burke C (2018). Mycophenolate-induced colitis. Am J Gastroenterol.

[7] Cleveland NK, Kinnucan J, Hart J, Rubin D (2015). A diagnostic dilemma: diarrhea and dermatitis. Am J Gastroenterol.

[8] Sondhi AR, Nayak LJ, Rice MD (2020). A case of unremitting diarrhea. Gastroenterology.

[9] Krishnamurthi SS, Kamath S (2022). UpToDate. Chemotherapy-Associated Diarrhea, Constipation and Intestinal Perforation: Pathogenesis, Risk Factors, and Clinical Presentation.

